# Seasonal Changes in Performance Metrics, Hormonal, Hematological, and Biochemical Markers Among Semi-Professional Soccer Players: Implications for Training and Recovery

**DOI:** 10.3390/jfmk10020147

**Published:** 2025-04-27

**Authors:** Eleftherios Mylonis, Dimitrios I. Bourdas, Natalia Kompodieta, Athanasios Tegousis, Panteleimon Bakirtzoglou, Athanasios Souglis, Evangelos Bekris

**Affiliations:** 1 School of Physical Education and Sports Science, Aristotle University of Thessaloniki, Ippokratous 22, Agios Ioannis, 62122 Serres, Greece; emylonis@phed-sr.auth.gr; 2 Section of Sport Medicine & Biology of Exercise, School of Physical Education and Sports Science, National and Kapodistrian University of Athens, 41 Ethnikis Antistasis, 17237 Athens, Greece; dbourdas@phed.uoa.gr; 3 School Physical Education and Sport Science, National and Kapodistrian University of Athens, Ethnikis Antistasis 41, 17237 Dafni, Greece; kompodieta.n@gmail.com (N.K.); thanasistegousis17@gmail.com (A.T.); asouglis@phed.uoa.gr (A.S.); vagbekris@phed.uoa.gr (E.B.); 4 School of Physical Education and Sport Science, Aristotle University of Thessaloniki, University Campus, 54124 Thessaloniki, Greece

**Keywords:** football, game, match, physical activity, sport, injury risk, indexes

## Abstract

**Objectives**: This study examined physiological, biochemical, and performance adaptations in 18 semi-professional male soccer players across three seasonal phases: pre-season initiation (PS), pre-competition (PC), and mid-season (MS). **Methods**: Assessments included physical/performance/hormonal/biochemical markers. **Results**: From PS to PC, body fat (Cohen’s *d* = −0.88; *p* ≤ 0.01) and speed drop rate (Cohen’s *d* = −1.52; *p* ≤ 0.01) significantly decreased, while V̇O_2_max (Cohen’s *d* = 0.80; *p* ≤ 0.01), velocity at V̇O_2_max (Cohen’s *d* = 1.86; *p* ≤ 0.01), and velocity at the second ventilatory threshold (Cohen’s *d* = 1.54; *p* ≤ 0.01) significantly increased. Significant fluctuations were observed in creatine kinase (Cohen’s *d* = 4.34; *p* ≤ 0.01), myoglobin (Cohen’s *d* = 0.66; *p* ≤ 0.01), and cortisol (Cohen’s *d* = −1.14; *p* ≤ 0.01) levels. From PS to MS, further reductions in body fat (Cohen’s *d* = −0.81; *p* ≤ 0.01) and speed drop rate (Cohen’s *d* = −1.12; *p* ≤ 0.01) were observed, along with significant improvements in countermovement jump performance (Cohen’s *d* = 1.08; *p* ≤ 0.01) and cardiorespiratory fitness (Cohen’s *d* ≥ 0.83; *p* ≤ 0.01). Creatine kinase (Cohen’s *d* = 3.82; *p* ≤ 0.01), myoglobin (Cohen’s *d* = 1.50; *p* ≤ 0.01), interleukin-6 (Cohen’s *d* = 1.24; *p* ≤ 0.01), and testosterone (Cohen’s *d* = 0.92; *p* ≤ 0.01) significantly increased. Stability in lower limb strength, flexibility, triglycerides, C-reactive protein, ferritin, liver enzymes, and most hematological parameters suggest resilience to seasonal demands. **Conclusions**: Seasonal training enhanced fitness and hormonal balance while maintaining physiological stability. These findings underscore the importance of periodized training to manage muscle damage and sustain an anabolic hormonal profile for peak performance. Consistent diet and training support metabolic health, while tailored recovery strategies and season-specific interventions are essential for optimizing performance and minimizing injury risk.

## 1. Introduction

Soccer requires high aerobic capacity, intermittent sprints, and rapid directional changes [1,2,3]. Performance depends on speed, flexibility, strength, and power [4,5,6,7], necessitating a balance between aerobic and anaerobic fitness [8]. Additionally, technical skills, tactics, anthropometrics, biochemical profiles, and training regimens play crucial roles in player performance [9,10].

The annual training macrocycle includes pre-season (~8 weeks, 7–10 training sessions/week) [11,12], competition (~35 weeks, ~6 training sessions/week, 1–2 official games) [13], and a transitional period. Training and matches induce physiological changes (e.g., performance, biochemical, hormonal, hematological) essential for assessing player status [10]. Evaluations at key season phases guide training strategies and optimize performance [14]. In this context, extensive research on soccer players has investigated physical, biochemical, and physiological attributes [15,16,17,18], including aerobic [19,20,21] and anaerobic capacities [22,23,24], somatometric characteristics [25,26,27,28], jumping ability [11,29,30], leg muscle strength [31,32], speed [33,34], agility, and flexibility [35] across various player demographics [16,36,37,38,39,40,41], and skill levels [19,20]. However, existing research mainly examines the immediate effects of matches or training on performance, biochemical, hormonal, and muscle damage markers [42,43,44,45,46], with limited studies addressing long-term variations [33,47,48]. A gap remains in assessing key physiological and performance adaptations in semi-professional soccer players across the pre-season, competition onset, and mid-season.

Optimizing soccer performance involves a complex interplay of numerous intrinsic and extrinsic factors influencing match outcomes [49]. Continuous monitoring of physical, biochemical, hormonal, and performance markers over extended periods, such as from pre-season preparation phase to mid-season, offers coaches insights into potential performance declines before match setbacks occur [50]. Yet, a research gap exists in examining long-term changes in vital markers among semi-professional male soccer players. Our study aims to bridge this gap by investigating physical, hematological, hormonal, biochemical, and performance indicators in semi-professional soccer players throughout the initiation of the pre-season preparation phase to the end of the pre-season preparation phase (i.e., the initiation of the competition phase) and to the mid-season period (i.e., the end of the first round of the competitive phase). It was hypothesized that physical, biochemical, hormonal, and performance markers would exhibit significant variations across extended periods, encompassing pre-season preparation, pre-competition, and mid-season.

## 2. Materials and Methods

### 2.1. Participants

**Figure 1:**
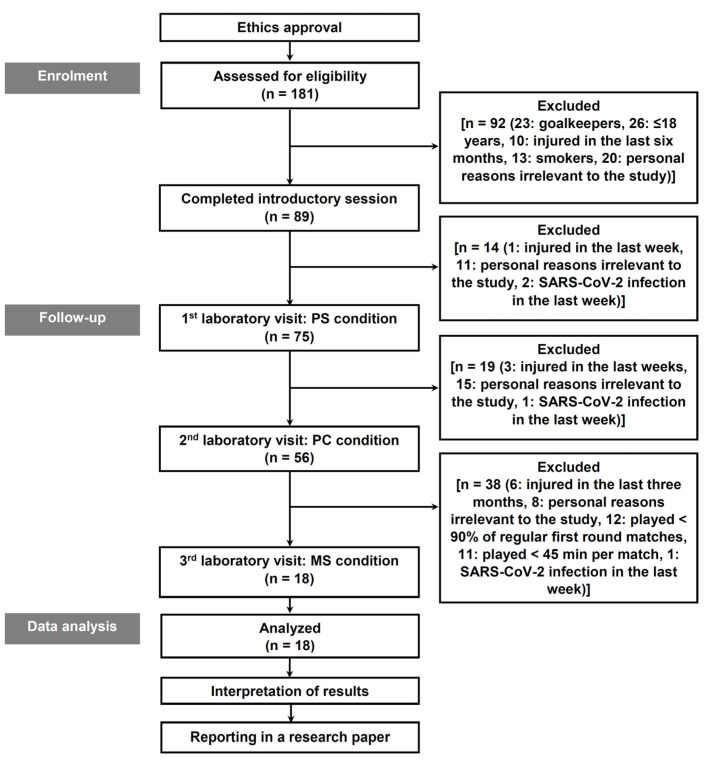
Research design. Abbreviations—PS: initiation of the pre-season preparation phase; PC: initiation of the competition phase (i.e., prior to the first official game of the season); MS: mid-season (i.e., post-first round break); n: sample size of the group.

Participants provided written informed consent after comprehensive disclosure regarding the study’s objectives, procedures, potential benefits, and risks. Before commencement (Figure 1), potential participants completed medical assessments and questionnaires on smoking habits, sleep patterns, and physical activity, drawing from validated sources [51,52,53,54]. Inclusion criteria encompassed male, highly active individuals (>1000 MET-min·week^−1^) [55], aged over 18, non-smoker, highly trained/national-level soccer players (defender, midfielder, attacker), devoid of injury, medication, or alcohol use for six months, living below 1500 m altitude, and exclusive focus on soccer during the study. Participants were required to have played a minimum of 90% of official matches (>45 min per match) as defenders, midfielders, or attackers. All procedures adhered to the Declaration of Helsinki guidelines [56] and received approval from the Local Ethics Research Committee of the School of Physical Education and Sport Science at Serres, Aristotle University of Thessaloniki, Greece (ERC-002/2023, approved on 2 October 2023).

Recruitment targeted male third National Division Greek Football players (16 clubs), with 351 registered individuals informed about the study. Of the 181 interested players, 163 were excluded either due to non-compliance with inclusion criteria or personal withdrawal unrelated to the study (Figure 1). Ultimately, 18 soccer players (tier 3, [57]: 6-defenders, 7-midfielders, 5-attackers) meeting the criteria (age = 25.00 ± 5.05 yr, height = 180.77 ± 5.93 cm, body mass = 78.51 ± 5.25 kg, body fat = 10.50 ± 1.67%, experience in soccer = 7.22 ± 3.63 yr, soccer-related training = 9.82 ± 1.33 h·wk^−1^) comprised the study cohort.

### 2.2. Procedures

**Figure 2:**
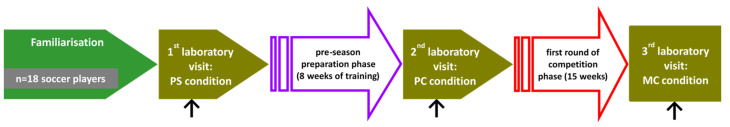
Experimental procedure: diagram showing the time course of assessments. Abbreviations—PS: initiation of the pre-season preparation phase; PC: initiation of the competition phase (i.e., prior to the first official game of the season); MS: mid-season (i.e., post-first round break); n: sample size of the group; ↑: physical, hematological, hormonal, biochemical, and performance assessments.

One week prior to the study commencement, participants visited the laboratory for familiarization with the testing protocol (Figure 2). Based on empirical evidence from the EROS study [58] and their relevance to the researchers [15], a series of physical, hematological, hormonal, biochemical, and performance tests were conducted across three distinct examination days: one day prior to the initiation of the pre-season preparation phase (PS—July), after eight weeks and approximately one week prior to the initiation of the competition phase (PC—September, i.e., prior to the first official game of the season), and approximately after fifteen weeks, that is, mid-season (MS—December, i.e., post-first round break of the competition phase). All examination days occurred at least 6 days after a friendly or official match.

Before the initial condition (i.e., PS), participants were instructed to follow a balanced diet (50–60% carbohydrates, 25–30% fat, and 15% protein) and meticulously record dietary intake. This dietary regimen was replicated in the weeks preceding the subsequent conditions (i.e., PC and MS). On the evening before examination days, participants consumed a carbohydrate-rich dinner (100 g plain pasta, 180 g grilled chicken breast, and a medium-sized banana) comprising approximately 65% of total energy intake [15].

In all conditions, participants fasted overnight and underwent examinations between 9:00 and 11:00 to minimize chronobiological effects. They were advised against consuming ergogenic substances [59,60,61,62] for one week before examination days. Players maintained regular nutrition, refrained from alcohol or medications, were in good health, resided at consistent altitudes, and adhered to regular sleep patterns. Forty-eight hours before testing, participants avoided vigorous exercise and engaged solely in low-load training sessions such as tactical practice or team cohesion drills.

Participants were instructed to perform at their best without feedback until the study’s conclusion. They remained unaware of their performance and refrained from discussing the study to prevent any external influence. Both participants and assessors were unaware of the research’s true objectives (double-blinded design).

During each laboratory visit, all assessments were performed sequentially with 10-min intervals between tests, except for the maximal oxygen uptake measurement, which was conducted following a 30 min recovery period to ensure adequate participant recuperation, as detailed later in the manuscript. The same-day administration of multiple tests was implemented primarily due to team scheduling constraints and logistical limitations during the competitive season. Additionally, this approach helped minimize the variability associated with external factors such as circadian rhythms, dietary intake, and training loads, thereby ensuring more standardized and controlled testing conditions across all time points. Tests were conducted in a standardized laboratory setting (temperature 22–25 °C, humidity 55–65%). Prior to each test, instruments were calibrated per manufacturer specifications. Participants emptied their bladders before performance tests and had access to water ad libitum.

### 2.3. Measurements

#### 2.3.1. Anthropometrics

Body mass was assessed using a Beam balance 710 (Seca, Birmingham, UK), recorded to the nearest 0.01 kg. Height measurements were taken using a Stadiometer^®^ (Seca, Birmingham, UK). Skinfold thickness at four sites (biceps, triceps, subscapularis, and iliacus muscle) was measured using a skinfold caliper (Harpenden Skinfold Calipers, Baty International, West Sussex, UK), and body fat percentage was estimated using Durnin and Womersley’s equations (body fat (%) = ((4.95/density) − 4.5) × 100; density = c − m × log skinfold thickness; c = 1.1620, or 1.1631, or 1.1422 for 18–19 yr, 20–29 yr, and 30–39 yr, respectively; and m = 0.0630, or 0.0632, or 0.0544 for 18–19 yr, 20–29 yr, and 30–39 yr, respectively [63,64]). Two measurements were made, and the average was noted. The median value was utilized in a third measurement if differences were found to be greater than 0.4 g for body mass, 4 mm for height, and 2 mm for skinfolds.

#### 2.3.2. Blood Sampling and Assays

Blood samples were collected at a consistent time of day, with participants seated, following an overnight fast. Samples were drawn from either the basilic or mesobasilic vein. For analysis of red blood cell count, hemoglobin, hematocrit, iron, glucose, cholesterol, and triglyceride levels, 10 μL of blood was introduced into dedicated test kits (Mini-Cuvettes: LKM/142/143/144/130/141/226/227, respectively; Dr. Lange, Hamburg, Germany) and analyzed using a spectrophotometer (Miniphotometer Plus LP20, Dr. Lange, Hamburg, Germany). White blood cell and platelet counts were assessed using 10 mL of blood collected into tubes containing EDTA K3 anticoagulant and processed on a Beckman Coulter Counter MAXM system (Beckman Coulter, Inc., Indianapolis, IN, USA). Serum testosterone levels were quantified using the ADVIA Centaur XP immunoassay platform (Siemens, Malvern, PA, USA). Cortisol and interleukin-6 concentrations were determined through enzyme-linked immunosorbent assays (E-EL-0157 and E-EL-H6156 ELISA kits, respectively; Elabscience, Houston, TX, USA) according to the manufacturer’s protocols, using a standard ELISA reader (Spark 10M^®^, Tecan, Männedorf, Switzerland). C-reactive protein levels were measured via immunonephelometry on a COBAS e411^®^ analyzer (F. Hoffmann-La Roche Ltd., Rotkreuz, Switzerland) following the manufacturer’s instructions. Creatine kinase activity was determined with Roche CK test strips^®^ and a Reflotron Plus System^®^ reflectance photometer (Roche, Basel, Switzerland). The activities of serum glutamic-oxaloacetic transaminase and serum glutamic pyruvic transaminase were evaluated using the automated SMAC technique, standardized with SMAC Reference II reagents (Technicon Corp., Tarrytown, NY, USA). Myoglobin concentrations were assessed with the IA-100 compact immunoassay system (Sysmex Corporation, Kobe, Japan). For ferritin analysis, blood samples were collected in plain tubes, centrifuged at 2000 rpm for 10 min, and the serum was stored at −20 °C. Ferritin was quantified using a radioimmunoassay kit (Bio-Rad Laboratories, Hemel Hempstead, UK). All measurements were performed in duplicate, with the average values used for statistical analysis. Based on the manufacturer’s internal validations, the coefficients of variation for intra-assay and inter-assay precision were approximately 2.4–8.7% and 3.5–8.9%, respectively.

#### 2.3.3. Flexibility of the Lower Back and Hamstrings

Following a standardized 10 min warm-up comprising low-intensity jogging and dynamic/static whole-body stretches, flexibility of the lower back and hamstrings assessment was conducted using a sit and reach box (Cranlea Human Performance Limited, Birmingham, UK) [65]. Participants, after removing their shoes, sat on the floor with legs extended straight ahead, placing the soles of their feet against the box. With knees locked and pressed flat to the ground, participants reached forward along the measuring line with palms facing downwards and hands stacked. Researchers recorded the maximum distance reached by both hands together among three trials that were separated by a one-minute rest period.

#### 2.3.4. Lower Limb Power and Strength

Following a standardized 5 min warm-up on a leg cycle ergometer (894E^®^, Monark, Varberg, Sweden) [41] at an intensity corresponding to 60–70% of the estimated maximal heart rate (HRmax), calculated using the HUNT formula (i.e., HRmax = 211 − 0.64 × age) [66], and performed at a self-selected cadence of 50–75 rpm, lower limb power was assessed via the countermovement jump (CMJ) with an arm swing from a standing position [67]. Jump performance was measured using an infrared contact timing platform (ERGO JUMP Plus—BOSCO SYSTEM, Byomedic, S.C.P., Barcelona, Spain). Participants executed three vertical jumps from a squat position at 90°, maintaining an upright body posture with hands positioned around the waist. The procedure was performed three times [68], separated by a one-minute rest period, and the maximum value was recorded.

Subsequently, maximal strength-power of the flexor-extensor knee was evaluated under isotonic-ballistic conditions for both legs (dominant and non-dominant). Using a dynamometer (Ergopower, Ergotest Technology A.S., Langesund, Norway), participants performed three maximal knee flexion and extension movements with a submaximal load set at 50% of their maximum, allowing for a twenty-second reset between trials. The highest recorded power value was used for statistical analysis.

#### 2.3.5. Running-Based Anaerobic Sprint Test

After a standardized 5 min warm-up involving low-intensity jogging and short intervals of high-intensity running, participants’ maximal speed was assessed using the Running-Based Anaerobic Sprint Test (RAST) [69] on an indoor small-sided soccer field (dimensions: 57 × 37 m) with artificial turf (Prestige XM6 60–13, FieldTurf^®^, Tarkett Sports, Paris, France). The test consisted of six maximal speed sprints covering 35 m each (designated by cones) with a 10 s break between repeats. Sprint times were accurately recorded using infrared photoelectric cells interfaced with a timing system (Saint Wien Digital Timer Press H5K, Lu-Chou City, Taipei Hsien, Taiwan) offering a time resolution of 0.01 s and a measurement error of ±0.01 s. RAST was chosen due to its established correlation with the Wingate test and its validated reliability in assessing anaerobic power, predicting short-distance performance, and evaluating fatigue [70]. However, rather than calculating traditional power-based metrics (e.g., peak and mean power, fatigue index), this study focused on two time-based variables: the average 35 m sprint time and the speed drop rate = ((slowest time − faster time)/faster time) × 100, which is an indicator analogous to the fatigue index. This approach was adopted to enhance ecological validity and field applicability, as these measures provide a more direct assessment of sprint capacity and fatigue resistance, are less affected by body mass variability, and are more robust to minor timing errors. Additionally, they offer greater interpretability and practicality for coaches and sport scientists in real-world soccer performance monitoring and training prescription.

#### 2.3.6. Maximal Oxygen Uptake, Lactate Concentration, and Heart Rate

Participants underwent an incremental running test to exhaustion on a motorized treadmill (Technogym Runrace^®^, Technogym, Gambettola, Italy) to determine maximal oxygen uptake (V̇O_2_max) utilizing gas exchange analyzers (OM-11 and LB-2 gas analyzers, Beckman Instruments, Inc., Indianapolis, IN, USA). The protocol commenced with a 5 min warm-up at 0% incline, followed by running at 7 km·h^−1^ for 1 min and 8 km·h^−1^ for 30 s. Subsequently, the treadmill speed increased by 0.5 km·h^−1^ every 30 s until exhaustion, with a 1% incline. Maximal oxygen uptake and HRmax were identified as the highest values achieved within 15 and 5 s intervals, respectively, during the final phase of the incremental test. The criteria for reaching V̇O_2_max were based on at least two of the following: a respiratory exchange ratio > 1.1, HRmax within 10 b·min^−1^ of estimated HRmax by age, a rating of perceived exertion equal to or exceeding 18, or a plateau in V̇O_2_ (<2 mL·kg^−1^·min^−1^) with an increase in treadmill speed [71]. Furthermore, the second ventilatory threshold (VT_2_) was identified using a consensus approach. Two blinded specialists independently assessed VT_2_ based on established criteria [72]. The VT_2_ determinations were then averaged and retained for further analysis. This approach provided an objective measure of VT_2_ expressed as both heart rate (HR) and velocity, serving as performance indicators for the participants.

Heart rate was telemetrically measured at 5 s intervals (Polar Sport tester, Polar Electro Oy, Kempele, Finland). Blood lactate concentrations were assessed with a portable analyzer (Lactate Plus, Nova Biomedica, Waltham, MA, USA) by applying a touch strip to a capillary blood sample (5–25 μL) collected from the left index finger 10 min after the completion of the V̇O_2_max protocol. Each sample was analyzed in duplicate, and the average value was used for subsequent statistical evaluation. According to the manufacturer’s internal validations, coefficients of variation for imprecision, reflecting both intra-assay and inter-assay variability, ranged from 3.4% to 5.9% for lactate measurements within the range from 2.6 to 10.5 mmol·L^−1^.

### 2.4. Statistical Analyses

The homogeneity and normality of the data were assessed using Levene’s and Shapiro-Wilk tests, respectively. An analysis of variance (ANOVA) with repeated measures on the condition factor was employed to evaluate the impact of conditions (PS, PC, and MS) on dependent variables. Mauchly’s test of sphericity was conducted, and when the assumption of sphericity was violated, Greenhouse-Geisser corrections were applied. For statistically significant interactions, Bonferroni post hoc pairwise comparisons were conducted. Additionally, effect sizes were calculated using Cohen’s d to assess the magnitude of pairwise differences (d values of 0.2, 0.5, and 0.8 were interpreted as small, medium, and large effects, respectively) [73,74]. Statistical analyses were performed using the Statistical Package for the Social Sciences for Windows (SPSS 27.0, IBM Corp, Armonk, NY, USA) following procedures outlined by Meyers et al. [75]. The threshold for statistical significance was set at *p* < 0.05. Unless otherwise noted, data are displayed as means ± standard deviations (SDs), with 95% confidence intervals (CIs) enclosed in square brackets.

Furthermore, a post hoc power analysis was performed using GraphPad StatMate Version 2.0 software (GraphPad Software Inc., La Jolla, CA, USA) to ensure the adequacy of the sample size for the study design. The analysis focused on V̇O_2_max as the primary variable, employing an effect size ($\eta_{p}^{2}$) of 0.83, an alpha level (α) of 0.05, a sample size of 18 participants, and a within-subjects design with three conditions (PS, PC, and MS). The observed power (1 − β) was calculated to be above 0.85. Similar power values were obtained when other key variables, such as body fat percentage, speed drop rate, velocity at V̇O_2_max (vV̇O_2_max), cholesterol, creatine kinase, myoglobin, iron, and interleukin-6, were included in the analysis.

## 3. Results

**Figure 3:**
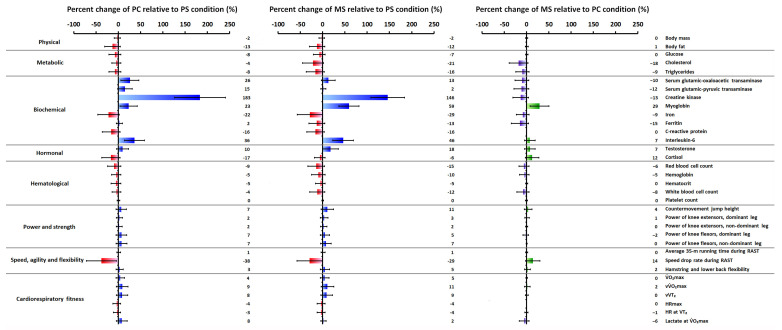
Depiction of percentage changes in mean values for physical, metabolic, biochemical, hormonal, hematological, and performance parameters from PS to PC (**left**), from PS to MS (**center**), and from PC to MS (**right**). Error bars present the lower and upper bounds of the 95% CI. Abbreviations—CI: confidence interval; HR: heart rate; HRmax: maximal heart rate; RAST: running-based anaerobic sprint test; PS: initiation of the pre-season preparation phase; PC: initiation of the competition phase (i.e., prior to the first official game of the season); MS: mid-season (i.e., post-first round break); V̇O_2_max: maximal oxygen uptake; VT_2_: second ventilatory threshold; vV̇O_2_max: velocity at V̇O_2_max; vVT_2_: velocity at VT_2_.

Table 1 displays the physical data; Table 2 displays the metabolic, biochemical, and hormonal data; Table 3 displays the hematological data; Table 4 displays the power, strength, speed, agility, and hamstring and lower back flexibility data; and Table 5 displays the cardiorespiratory fitness data for PS, PC, and MS conditions, respectively. Detailed results of the Bonferroni-adjusted post hoc comparisons (PS-PC, PS-MS, and PC-MS), including exact *p*-values and corresponding Cohen’s d effect sizes with corresponding 95% CIs for pairwise comparisons between conditions (PS-PC, PS-MS, and PC-MS) across all variables, are provided in Tables S1 and S2 (see Supplementary Materials), respectively. Moreover, Figure 3 illustrates the percentage changes in all parameters from PS to PC, from PS to MS, and from PC to MS. Briefly, the period from the PS to the PC condition was marked by significant changes (*p* ≤ 0.05), including reductions in body fat (−12.86 ± 0.38% [−30.45–4.74]) and speed drop rate (−37.96 ± 0.72% [−71.39–−4.53]), alongside significant improvements (*p* ≤ 0.05) in physiological and performance metrics such as V̇O_2_max (+4.43 ± 0.21% [−5.08–13.94]), vV̇O_2_max (+8.81 ± 0.28% [−4.28–21.91]), and vVT_2_ (+8.35 ± 0.28% [−4.43–21.13]). Concurrently, substantial fluctuations (*p* ≤ 0.05) were noted in white blood cell count (−4.32 ± 0.21% [−14.13–5.49]) and in biochemical markers, creatine kinase (+183.48 ± 1.24% [126.31–240.66]), myoglobin (+23.10 ± 0.42% [3.63–42.57]), and cortisol (−16.55 ± 0.44% [−36.84–3.74]) were noted. From the PS to the MS condition, a significant reduction (*p* ≤ 0.05) in body fat (−12.38 ± 0.37% [−29.61–4.85]) and speed drop rate (−29.20 ± 0.61% [−57.57–−0.82]) was accompanied by significant enhancements (*p* ≤ 0.05) in countermovement jump performance (+10.5 ± 0.31% [−3.66–24.67]), V̇O_2_max (+4.57 ± 0.21% [−5.08–14.23]), vV̇O_2_max (+10.76 ± 0.31% [−3.55–25.08]), and vVT_2_ (+8.88 ± 0.28% [−4.26–22.02]). This condition also exhibited pronounced alterations in white blood cell count (−12.03 ± 0.37% [−28.99–4.93]), biochemical and hormonal parameters, including significant changes in creatine kinase (+146.21 ± 0.82% [108.24–184.18]), myoglobin (+59.1 ± 0.49% [36.39–81.81]), interleukin-6 (+46.08 ± 0.5% [23.05–69.11]), testosterone (+17.68 ± 0.38% [0.05–35.3]), and cortisol (−6.29 ± 0.26% [−18.24–5.66]). In contrast, several markers—lower limb strength, hamstring and lower back flexibility, triglycerides, C-reactive protein, ferritin, serum glutamic-oxaloacetic transaminase, serum glutamic-pyruvic transaminase, red blood cell count, hemoglobin, and hematocrit—remained stable throughout the study period.

## 4. Discussion

This study evaluated seasonal variations in physiological, biochemical, and performance indicators among semi-professional male soccer players. Results demonstrated significant improvements in cardiorespiratory fitness and marked fluctuations in creatine kinase, myoglobin, testosterone, and cortisol, reflecting adaptive responses to varying training loads and competitive demands. Stable markers, such as C-reactive protein and ferritin, indicate maintained inflammatory and iron equilibrium, while consistent glucose and lipid profiles underscore the positive impact of a well-regulated dietary and training regimen on metabolic health.

Transition periods are known to adversely affect physical and skill performance in the absence of targeted training programs [76,77]. Conversely, high-load pre-season preparation restores or enhances fitness [78,79], with competition phases emphasizing performance maintenance through structured micro- and mesocycles [14]. Consistent with prior findings [26,28], this study observed significant body fat reductions across all phases, attributed to training intensity and dietary adjustments [25], while body mass remained stable.

Explosive leg strength, critical for soccer performance [14], improved significantly, particularly in vertical jump height, aligning with reports of pre- and mid-season gains [11,80]. Strength training [81] and systematic plyometric interventions [48] likely drove these improvements. Notably, muscle strength showed no bilateral asymmetry, minimizing injury risks [82,83] and performance inefficiencies [84]. Hamstring and lower back flexibility displayed minor changes but remained largely stable throughout the study. Speed-related outcomes revealed consistent mean speed but improved speed endurance, evidenced by a reduced speed drop rate and enhanced V̇O_2_max, consistent with Bekris et al. [48]. These findings reflect the contrasting demands of preparation and competition phases and highlight the effectiveness of tailored conditioning protocols.

Soccer players typically exhibit V̇O_2_max values ranging from 51 to 62.9 mL·kg^−1^·min^−1^ [85], consistent with our findings. In this study, V̇O_2_max significantly improved across all conditions, mirroring trends reported in professional [11,20,22,40,80,86] and youth players [37,87]. These gains likely stem from aerobic-specific training during the pre-season [22] and physiological adaptations from match play [77]. The mean vV̇O_2_max also increased significantly from 16.91 ± 0.78 km·h^−1^ in the PS condition to 18.73 ± 0.67 km·h^−1^ in the MS condition, aligning with typical ranges of 13–17 km·h^−1^ for soccer players [88]. Consistent pre-season and mid-season improvements in the maximum aerobic speed observed here [20,37,87] are indicative of the cumulative benefits of aerobic training and match activity. Similarly, vVT_2_ improved significantly over the study period, consistent with findings in British and Greek players [22,40]. These gains could also be attributable to reductions in body fat, which enhance aerobic capacity and performance metrics [20,89].

Glucose levels remained stable across all conditions, consistent with prior research [90] indicating no seasonal changes in competitive athletes. This stability likely reflects adherence to a balanced diet. In contrast, cholesterol levels decreased significantly from PS to MS, mirroring findings in Indian football players [91] and suggesting enhanced fat metabolism during pre-season aerobic training. Stabilization mid-season may reflect sustained physical activity and training loads. Triglycerides, serum glutamic-oxaloacetic transaminase, and serum glutamic-pyruvic transaminase showed no significant changes, contrasting with studies reporting increases post-preparation [9,92]. This stability likely reflects structured training and balanced nutrition maintaining metabolic homeostasis throughout the season.

In some previous studies [48,93], no significant changes were observed in red blood cell count, hematocrit, hemoglobin levels, or platelet count. In this study, significant declines were observed in red blood cell count, hematocrit, and hemoglobin levels, aligning with some prior findings [94,95] possibly due to increased training demands or dissimilarities in nutrition/hydration, injury occurrences, and competition levels. White blood cell count declined significantly at the MS condition compared to PS and PC conditions. Similar fluctuations have been documented over training periods [47], while some studies report increases [96] or no changes [97]. Variability in white blood cell responses may stem from factors such as physiological stress, subclinical infections, or nutritional deficiencies during intense training [98,99,100]. Tailored recovery and nutritional protocols are critical to supporting immune function and performance under these demands.

C-reactive protein, a marker of systemic inflammation, is influenced by intense physical exercise [101]. In this study, no significant changes were observed across the assessment periods, contrasting with studies on professional players that reported increases during conditioning [102]. The discrepancy may stem from differences in training volume, recovery capabilities, and individual factors such as genetics, fitness, nutrition, and sleep. These results suggest that semi-professional players may experience distinct inflammatory responses, warranting further research into how training intensity and competition levels influence long-term inflammation. Creatine kinase levels increased significantly at the PC and MS conditions compared to PS, consistent with prior studies linking rises to higher training loads and cumulative match participation [46,103,104]. This trend reflects muscle stress during conditioning and mid-season phases, corroborating findings in professional soccer players [105,106]. Myoglobin concentrations, a marker of muscle damage, showed progressive increases, with higher values at PC and MS compared to the PS condition. This pattern likely reflects cumulative muscle damage due to elevated physical demands during the competitive season, consistent with observations in soccer players [33]. Iron metabolism exhibited significant changes, with iron concentrations decreasing from PS to PC and MS conditions, while ferritin levels remained stable. This suggests heightened demands for iron during erythropoiesis and energy metabolism, leading to transient depletion despite sufficient iron stores, as reported in similar studies [48,80]. Insufficient dietary iron intake may exacerbate these demands, highlighting the need for nutritional strategies tailored to soccer players’ metabolic requirements.

Exercise-induced interleukin-6 (IL-6) elevation is a well-established response to maximal exercise [96], yet long-term studies on soccer players remain scarce. In this study, IL-6 concentrations were significantly higher at the PC and MS conditions compared to PS. This rise at the conclusion of the pre-season preparation phase reflects the high training volume and intensity aimed at enhancing endurance and strength [78,79]. Mid-season elevations are likely due to match play, which imposes repeated high-intensity efforts, causing muscle damage and triggering an inflammatory response essential for tissue repair and metabolic adaptations. These findings emphasize the dual role of IL-6 as both an inflammation marker and a mediator of physiological adaptations in soccer.

Long-term hormonal adaptations in soccer players are less explored compared to acute match-related responses [18,107]. Testosterone levels significantly increased from PS to MC, aligning with studies in professional players [103] and young high-level athletes [33,108]. This rise, alongside a marked decline in cortisol, suggest an anabolic environment conducive to muscle protein synthesis and recovery [86,108]. The hormonal shift likely supports adaptations to training demands and improved performance metrics such as vertical jump height [109,110], underscoring the connection between hormonal regulation and functional improvements in soccer players.

### Strengths, Limitations, and Future Research Directions

The findings of this study are specific to adult male soccer players, limiting applicability to other groups. While participants were presumed to have similar schedules and training regimens, individual variations in training load, fatigue, and unmonitored dietary habits may have influenced the outcomes. Another methodological limitation of this study concerns the same-day administration of physical tests engaging different energy systems, such as the RAST (anaerobic) and V̇O_2_max (aerobic) protocols. Although tests were spaced with recovery intervals (10 min between assessments; 30 min before V̇O_2_max), we recognize that complete metabolic recovery—particularly of anaerobic energy systems—may not have been fully achieved. This cumulative testing load could have influenced maximal effort capacity, representing a potential source of bias. Nonetheless, the decision to perform all assessments on the same day was based on logistical constraints, ethical considerations related to athlete availability during the competitive season, and the need to ensure standardized, within-day comparisons of physiological variables. Future studies might consider multi-day testing to more precisely isolate each performance domain. Furthermore, the absence of a (true) control group restricted the ability to isolate the effects of distinct training phases on physical, biochemical, hormonal, and performance metrics. Psychological factors such as performance expectations may have also influenced laboratory-based assessments, despite efforts to mitigate placebo effects.

Future research should address these limitations by including diverse populations (e.g., female athletes, various playing positions, or competitive tiers), larger sample sizes, long-term dietary monitoring, and control groups. Exploring additional markers, including psychological and immunological indicators, could deepen understanding and provide broader insights.

Despite the aforementioned limitations, this study’s longitudinal design captured seasonal physiological, biochemical, and hormonal adaptations, providing rare insights into semi-professional soccer players. Rigorous methodologies ensured reliable data, and the inclusion of multiple markers (e.g., interleukin-6, testosterone, creatine kinase, myoglobin, and iron status) offered a holistic perspective on performance and recovery. Real-world training and match scenarios enhanced ecological validity, contributing novel insights into the interactions between training phases, competitive demands, and player adaptations.

## 5. Conclusions

This study examined seasonal variations in physiological, biochemical, and performance markers among semi-professional male soccer players across three phases of the season: pre-season preparation, competition onset, and post-first round break. Notable improvements in cardiorespiratory fitness and significant changes in creatine kinase, myoglobin, testosterone, and cortisol levels were observed, reflecting adaptations to varying training loads and competitive demands. Stable markers such as C-reactive protein and ferritin reflect a potential equilibrium in inflammation and iron storage mechanisms under the monitored conditions.

These observations emphasize the importance of periodized training and recovery protocols to mitigate muscle damage and sustain an anabolic environment for performance enhancement. The stability of glucose and lipid profiles further highlights the beneficial impact of consistent dietary and training regimens on metabolic health. This study contributes valuable insights into the physiological demands of soccer and supports the development of evidence-based practices for optimizing training and recovery strategies in semi-professional players.

## Figures and Tables

**Table 1:** Physical data for PS, PC, and MS conditions, presented as M ± SD [95% CI].

Variables	PS	PC	MS
Body mass (kg)	78.51 ± 5.25 [76.08–80.94]	76.82 ± 4.75 [74.63–79.01]	77.06 ± 4.93 [74.78–79.34]
†‡ Body fat (%)	10.50 ± 1.67 [9.73–11.27]	9.15 ± 1.35 [8.53–9.77]	9.20 ± 1.51 [8.50–9.90]

† Significant difference between PS and PC conditions at *p* ≤ 0.05. ‡ Significant difference between PS and MS conditions at *p* ≤ 0.05. Abbreviations—CI: confidence interval; M: mean; PS: initiation of the pre-season preparation phase; PC: initiation of the competition phase (i.e., prior to the first official game of the season); MS: mid-season (i.e., post-first round break); SD: standard deviation.

**Table 2:** Metabolic, biochemical, and hormonal data for PS, PC, and MS conditions, presented as M ± SD [95% CI].

Variables	PS	PC	MS
Glucose (mg·dL^−1^)	95.30 ± 20.48 [85.84–104.76]	87.94 ± 6.07 [85.14–90.74]	88.22 ± 7.01 [84.98–91.46]
‡§ Cholesterol (mg·dL^−1^)	190.80 ± 44.40 [170.29–211.31]	182.30 ± 36.99 [165.21–199.39]	150.00 ± 32.03 [135.20–164.80]
Τriglycerides (mg·dL^−1^)	91.44 ± 33.22 [76.09–106.79]	84.27 ± 37.92 [66.75–101.79]	76.38 ± 30.05 [62.50–90.26]
Serum glutamic-oxaloacetic transaminase (U·L^−1^)	26.38 ± 5.34 [23.91–28.85]	33.22 ± 12.14 [27.61–38.83]	29.77 ± 7.28 [26.41–33.13]
Serum glutamic-pyruvic transaminase (U·L^−1^)	23.33 ± 7.19 [20.01–26.65]	26.83 ± 10.54 [21.96–31.70]	23.72 ± 8.62 [19.74–27.70]
†‡§ Creatine kinase (U·L^−1^)	211.61 ± 75.80 [176.59–246.63]	599.88 ± 98.80 [554.24–645.52]	521.00 ± 85.20 [481.64–560.36]
†‡§ Myoglobin (mg·L^−1^)	50.00 ± 20.13 [40.70–59.30]	61.55 ± 9.97 [56.94–66.16]	79.55 ± 19.17 [70.69–88.41]
†‡ Iron (μg·dL^−1^)	114.38 ± 28.28 [101.32–127.44]	89.33 ± 26.03 [77.30–101.36]	81.61 ± 19.24 [72.72–90.50]
Ferritin (mg·L^−1^)	71.45 ± 34.55 [55.49–87.41]	73.03 ± 39.01 [55.01–91.05]	62.08 ± 29.70 [48.36–75.80]
C-reactive protein (mg·L^−1^)	0.44 ± 0.46 [0.23–0.65]	0.37 ± 0.10 [0.32–0.42]	0.37 ± 0.10 [0.32–0.42]
†‡ Interleukin-6 (mg·L^−1^)	8.16 ± 2.41 [7.05–9.27]	11.12 ± 3.39 [9.55–12.69]	11.92 ± 3.42 [10.34–13.50]
‡ Testosterone (ng·mL^−1^)	5.94 ± 1.11 [5.43–6.45]	6.51 ± 1.44 [5.84–7.18]	6.99 ± 1.17 [6.45–7.53]
† Cortisol (μg·dL^−1^)	18.91 ± 1.69 [18.13–19.69]	15.78 ± 3.17 [14.32–17.24]	17.72 ± 3.06 [16.31–19.13]

† Significant difference between PS and PC conditions at *p* ≤ 0.05. ‡ Significant difference between PS and MS conditions at *p* ≤ 0.05. § Significant difference between PC and MS conditions at *p* ≤ 0.05. Abbreviations—CI: confidence interval; M: mean; PS: initiation of the pre-season preparation phase; PC: initiation of the competition phase (i.e., prior to the first official game of the season); MS: mid-season (i.e., post-first round break); SD: standard deviation.

**Table 3:** Hematological data for PS, PC, and MS conditions, presented as M ± SD [95% CI].

Variables	PS	PC	MS
†‡ Red blood cells count (10^12^RBC·L^−1^)	6.73 ± 0.93 [6.30–7.16]	6.10 ± 1.13 [5.58–6.62]	5.75 ± 0.94 [5.32–6.18]
†‡§ Hemoglobin (g·dL^−1^)	15.56 ± 0.72 [15.23–15.89]	14.80 ± 0.88 [14.39–15.21]	14.06 ± 0.84 [13.67–14.45]
†‡ Hematocrit (%)	45.95 ± 2.05 [45.00–46.90]	43.46 ± 2.46 [42.32–44.60]	43.58 ± 1.88 [42.71–44.45]
†‡§ White blood cell count (10^9^WBC·L^−1^)	5.32 ± 0.22 [5.22–5.42]	5.09 ± 0.31 [4.95–5.23]	4.68 ± 0.35 [4.52–4.84]
Platelet count (10^9^PLT·L^−1^)	227.70 ± 29.58 [214.03–241.37]	228.61 ± 26.59 [216.33–240.89]	228.05 ± 26.54 [215.79–240.31]

† Significant difference between PS and PC conditions at *p* ≤ 0.05. ‡ Significant difference between PS and MS conditions at *p* ≤ 0.05. § Significant difference between PC and MS conditions at *p* ≤ 0.05. Abbreviations—CI: confidence interval; M: mean; PS: initiation of the pre-season preparation phase; PC: initiation of the competition phase (i.e., prior to the first official game of the season); MS: mid-season (i.e., post-first round break); SD: standard deviation.

**Table 4:** Power, strength, speed, agility, and hamstring and lower back flexibility data for PS, PC, and MS conditions, presented as M ± SD [95% CI].

Variables	PS	PC	MS
‡ Countermovement jump height (cm)	39.23 ± 4.12 [37.33–41.13]	41.84 ± 3.82 [40.08–43.60]	43.35 ± 3.36 [41.80–44.90]
Power of knee extensors, dominant leg (watt)	240.60 ± 29.92 [226.78–254.42]	246.20 ± 31.28 [231.75–260.65]	248.90 ± 26.37 [236.72–261.08]
Power of knee extensors, non-dominant leg (watt)	252.70 ± 28.92 [239.34–266.06]	258.00 ± 29.91 [244.18–271.82]	258.80 ± 27.68 [246.01–271.59]
Power of knee flexors, dominant leg (watt)	164.80 ± 21.26 [154.98–174.62]	176.50 ± 19.50 [167.49–185.51]	173.38 ± 16.53 [165.74–181.02]
Power of knee flexors, non-dominant leg (watt)	160.60 ± 28.48 [147.44–173.76]	172.30 ± 27.04 [159.81–184.79]	172.44 ± 23.17 [161.74–183.14]
Average 35 m running time during RAST (s)	4.74 ± 0.15 [4.67–4.81]	4.78 ± 0.15 [4.71–4.85]	4.79 ± 0.14 [4.73–4.85]
†‡ Speed drop rate during RAST (%)	13.70 ± 3.74 [11.97–15.43]	8.50 ± 3.01 [7.11–9.89]	9.70 ± 3.34 [8.16–11.24]
Hamstring and lower back flexibility (cm)	24.00 ± 6.63 [20.94–27.06]	24.77 ± 6.62 [21.71–27.83]	25.27 ± 6.04 [22.48–28.06]

† Significant difference between PS and PC conditions at *p* ≤ 0.05. ‡ Significant difference between PS and MS conditions at *p* ≤ 0.05. Abbreviations—CI: confidence interval; M: mean; RAST: running-based anaerobic sprint test; PS: initiation of the pre-season preparation phase; PC: initiation of the competition phase (i.e., prior to the first official game of the season); MS: mid-season (i.e., post-first round break); SD: standard deviation.

**Table 5:** Cardiorespiratory fitness data for PS, PC, and MS conditions, presented as M ± SD [95% CI].

Variables	PS	PC	MS
†‡ V̇O_2_max (mL·kg^−1^·min^−1^)	56.18 ± 3.37 [54.62–57.74]	58.67 ± 2.79 [57.38–59.96]	58.75 ± 2.67 [57.52–59.98]
†‡ vV̇O_2_max (km·h^−1^)	16.91 ± 0.78 [16.55–17.27]	18.40 ± 0.82 [18.02–18.78]	18.73 ± 0.67 [18.42–19.04]
†‡ vVT_2_ (km·h^−1^)	13.29 ± 0.74 [12.95–13.63]	14.40 ± 0.69 [14.08–14.72]	14.47 ± 0.69 [14.15–14.79]
†‡ HRmax (b·min^−1^)	197.00 ± 11.36 [191.75–202.25]	190.00 ± 11.76 [184.57–195.43]	190.00 ± 11.06 [184.89–195.11]
‡ HR at VT_2_ (b·min^−1^)	168.00 ± 9.99 [163.38–172.62]	163.00 ± 10.39 [158.2–167.80]	162.00 ± 9.74 [157.50–166.50]
Lactate at V̇O_2_max (mmol·L^−1^)	11.78 ± 1.68 [11.00–12.56]	12.68 ± 1.53 [11.97–13.39]	11.98 ± 1.54 [11.27–12.69]

† Significant difference between PS and PC conditions at *p* ≤ 0.05. ‡ Significant difference between PS and MS conditions at *p* ≤ 0.05. Abbreviations—CI: confidence interval; HR: heart rate; HRmax: maximal heart rate; M: mean; PS: initiation of the pre-season preparation phase; PC: initiation of the competition phase (i.e., prior to the first official game of the season); MS: mid-season (i.e., post-first round break); SD: standard deviation; V̇O_2_max: maximal oxygen uptake; VT_2_: second ventilatory threshold; vV̇O_2_max: velocity at V̇O_2_max; vVT_2_: velocity at VT_2_.

## Data Availability

The raw data supporting the conclusions of this article will be made available by the corresponding author upon reasonable request once all relevant subsidies are reported and completed.

## Supplementary Materials

The following supporting information can be downloaded at https://www.mdpi.com/article/10.3390/jfmk10020147/s1. Table S1: Bonferroni-adjusted *p*-values for pairwise comparisons between conditions (PS-PC, PS-MS, and PC-MS) across all variables; Table S2: Cohen’s d effect sizes and 95% confidence intervals, enclosed in square brackets, for pairwise comparisons between conditions (PS-PC, PS-MS, and PC-MS) across all variables.
