# Supplementary material for: Seasonal Changes in Performance Metrics, Hormonal, Hematological, and Biochemical Markers Among Semi-Professional Soccer Players: Implications for Training and Recovery
Source: J Funct Morphol Kinesiol. 2025 Apr 27;10(2):147. doi: 10.3390/jfmk10020147 (PMC12101407; doi:10.3390/jfmk10020147)
Supplement: Supplementary file 1 [file jfmk-10-00147-s001.zip › Tables S1.pdf]

Table S1. Bonferroni-adjusted p-values for pairwise comparisons between conditions (PS-PC, PS-MS, and PC-MS) across all variables.

Variables	PS-PC	PS-MS	PC-MS
Body mass	0.167	0.250	0.835
†‡Body fat	0.002	0.001	0.907
Glucose	0.108	0.120	0.867
‡§Cholesterol	0.385	0.001	0.001
Triglycerides	0.408	0.060	0.343
Serum glutamic-oxaloacetic transaminase	0.059	0.091	0.179
Serum glutamic pyruvic transaminase	0.125	0.83	0.192
†‡§Creatine kinase	0.001	0.001	0.002
†‡§Myoglobin	0.012	0.001	0.001
†‡Iron	0.001	0.001	0.179
Ferritin	0.867	0.240	0.206
C-reactive protein	0.481	0.480	0.998
†‡Interleukin-6	0.001	0.001	0.343
‡Testosterone	0.079	0.001	0.145
†Cortisol	0.001	0.070	0.018
†‡Red blood cells count	0.019	0.001	0.179
†‡§Hemoglobin	0.001	0.001	0.002
†‡Hematocrit	0.001	0.001	0.835
†‡§White blood cell count	0.003	0.001	0.001
Platelets count	0.900	0.970	0.933
‡Countermovement jump height	0.056	0.001	0.093
Power of knee extensors, dominant leg	0.456	0.240	0.707
Power of knee extensors, nondominant leg	0.456	0.360	0.901
Power of knee flexors, dominant leg	0.087	0.080	0.481
Power of knee flexors, nondominant leg	0.093	0.070	0.967
Average 35-m running time during RAST	0.268	0.180	0.770
†‡Speed drop rate during RAST	0.001	0.001	0.125
Hamstring and lower back flexibility	0.617	0.410	0.738
†‡$\dot{V}O_2$max	0.003	0.001	0.911
†‡v$\dot{V}O_2$max	0.001	0.001	0.086
†‡vVT_2	0.001	0.001	0.677
†‡HRmax	0.019	0.020	0.998
‡HR at VT_2	0.053	0.020	0.677
Lactate at $\dot{V}O_2$max	0.060	0.620	0.073

† Significant difference between PS and PC conditions at $p \leq 0.05$.

‡ Significant difference between PS and MS conditions at $p \leq 0.05$.

§ Significant difference between PC and MS conditions at $p \leq 0.05$.

Abbreviations—HR: heart rate; HRmax: maximal heart rate; M: mean; RAST: running based anaerobic sprint test; PS: initiation of the pre-season preparation phase; PC: initiation of the competition phase (i.e., prior to the first official game of the season); MS: mid-season (i.e., post-first round break); SD: standard deviation; $\dot{V}O_2$max: maximal oxygen uptake; VT_2: second ventilatory threshold; v$\dot{V}O_2$max: velocity at $\dot{V}O_2$max; vVT_2: velocity at VT_2.
