# Supplementary material for: Seasonal Changes in Performance Metrics, Hormonal, Hematological, and Biochemical Markers Among Semi-Professional Soccer Players: Implications for Training and Recovery
Source: J Funct Morphol Kinesiol. 2025 Apr 27;10(2):147. doi: 10.3390/jfmk10020147 (PMC12101407; doi:10.3390/jfmk10020147)
Supplement: Supplementary file 1 [file jfmk-10-00147-s001.zip › Tables S2.pdf]

Table S2. Cohen's d effect sizes and 95% CIs, enclosed in square brackets, for pairwise comparisons between conditions (PS-PC, PS-MS, and PC-MS) across all variables.

Variables	PS-PC	PS-MS	PC-MS
Body mass	-0.34 [-0.81 – 0.13]	-0.28 [-0.75 – 0.19]	0.05 [-0.42 – 0.52]
†‡Body fat	-0.88 [-1.43 – -0.33]	-0.81 [-1.34 – -0.28]	0.03 [-0.44 – 0.5]
Glucose	-0.4 [-0.87 – 0.07]	-0.39 [-0.86 – 0.08]	0.04 [-0.43 – 0.51]
‡§Cholesterol	-0.21 [-0.68 – 0.26]	-1.03 [-1.60 – -0.46]	-0.93 [-1.48 – -0.38]
Triglycerides	-0.20 [-0.67 – 0.27]	-0.47 [-0.96 – 0.02]	-0.23 [-0.70 – 0.24]
Serum glutamic-oxaloacetic transaminase	0.65 [0.14 – 1.16]	0.52 [0.03 – 1.01]	-0.33 [-0.80 – 0.14]
Serum glutamic pyruvic transaminase	0.38 [-0.09 – 0.85]	0.05 [-0.42 – 0.52]	-0.32 [-0.79 – 0.15]
†‡§Creatine kinase	4.34 [2.85 – 5.83]	3.82 [2.49 – 5.15]	-0.85 [-1.40 – -0.30]
†‡§Myoglobin	0.66 [0.15 – 1.17]	1.50 [0.83 – 2.17]	1.08 [0.49 – 1.67]
†‡Iron	-0.92 [-1.47 – -0.37]	-1.31 [-1.94 – -0.68]	-0.33 [-0.80 – 0.14]
Ferritin	0.04 [-0.43 – 0.51]	-0.29 [-0.76 – 0.18]	-0.31 [-0.78 – 0.16]
C-reactive protein	-0.17 [-0.64 – 0.30]	-0.17 [-0.64 – 0.30]	0.00 [-0.47 – 0.47]
†‡Interleukin-6	0.98 [0.41 – 1.55]	1.24 [0.63 – 1.85]	0.23 [-0.24 – 0.70]
‡Testosterone	0.44 [-0.05 – 0.93]	0.92 [0.37 – 1.47]	0.36 [-0.11 – 0.83]
†Cortisol	-1.14 [-1.73 – -0.55]	-0.45 [-0.94 – 0.04]	0.62 [0.11 – 1.13]
†‡Red blood cells count	-0.61 [-1.12 – -0.10]	-1.04 [-1.61 – -0.47]	-0.33 [-0.80 – 0.14]
†‡§Hemoglobin	-0.94 [-1.49 – -0.39]	-1.90 [-2.66 – -1.14]	-0.86 [-1.41 – -0.31]
†‡Hematocrit	-1.09 [-1.68 – -0.50]	-1.20 [-1.81 – -0.59]	0.05 [-0.42 – 0.52]
†‡§White blood cell count	-0.82 [-1.35 – -0.29]	-2.06 [-2.88 – -1.24]	-1.24 [-1.85 – -0.63]
Platelets count	0.03 [-0.44 – 0.50]	0.01 [-0.46 – 0.48]	-0.02 [-0.49 – 0.45]
‡Countermovement jump height	0.66 [0.15 – 1.17]	1.08 [0.49 – 1.67]	0.42 [-0.07 – 0.91]
Power of knee extensors, dominant leg	0.18 [-0.29 – 0.65]	0.29 [-0.18 – 0.76]	0.09 [-0.38 – 0.56]
Power of knee extensors, nondominant leg	0.18 [-0.29 – 0.65]	0.22 [-0.25 – 0.69]	0.03 [-0.44 – 0.50]
Power of knee flexors, dominant leg	0.57 [0.08 – 1.06]	0.44 [-0.05 – 0.93]	-0.17 [-0.64 – 0.30]
Power of knee flexors, nondominant leg	0.42 [-0.07 – 0.91]	0.45 [-0.04 – 0.94]	0.01 [-0.46 – 0.48]
Average 35-m running time during RAST	0.27 [-0.20 – 0.74]	0.33 [-0.14 – 0.80]	0.07 [-0.40 – 0.54]
†‡Speed drop rate during RAST	-1.52 [-2.21 – -0.83]	-1.12 [-1.71 – -0.53]	0.38 [-0.09 – 0.85]
Hamstring and lower back flexibility	0.12 [-0.35 – 0.59]	0.20 [-0.27 – 0.67]	0.08 [-0.39 – 0.55]
†‡VO_2max	0.80 [0.27 – 1.33]	0.83 [0.30 – 1.36]	0.03 [-0.44 – 0.50]
†‡vVO_2max	1.86 [1.10 – 2.62]	2.49 [1.55 – 3.43]	0.43 [-0.06 – 0.92]
†‡vVT_2	1.54 [0.85 – 2.23]	1.64 [0.93 – 2.35]	0.10 [-0.37 – 0.57]
†‡HRmax	-0.61 [-1.12 – -0.10]	-0.62 [-1.13 – -0.11]	0.00 [-0.47 – 0.47]
‡HR at VT_2	-0.49 [-0.98 – 0.00]	-0.61 [-1.12 – -0.10]	-0.10 [-0.57 – 0.37]
Lactate at VO_2max	0.56 [0.07 – 1.05]	0.12 [-0.35 – 0.59]	-0.45 [-0.94 – 0.04]

† Significant difference between PS and PC conditions at $p \leq 0.05$.

‡ Significant difference between PS and MS conditions at $p \leq 0.05$.

§ Significant difference between PC and MS conditions at $p \leq 0.05$.

Abbreviations—CI: confidence interval; HR: heart rate; HRmax: maximal heart rate; M: mean; RAST: running based anaerobic sprint test; PS: initiation of the pre-season preparation phase; PC: initiation of the competition phase (i.e., prior to the first official game of the season); MS: mid-season (i.e., post-first round break); SD: standard deviation; VO_2max: maximal oxygen uptake; VT_2: second ventilatory threshold; vVO_2max: velocity at VO_2max; vVT_2: velocity at VT_2.
